# Evaluation of CA125 in relation to pain symptoms among adolescents and young adult women with and without surgically-confirmed endometriosis

**DOI:** 10.1371/journal.pone.0238043

**Published:** 2020-08-24

**Authors:** Naoko Sasamoto, Mary DePari, Allison F. Vitonis, Marc R. Laufer, Stacey A. Missmer, Amy L. Shafrir, Kathryn L. Terry

**Affiliations:** 1 Boston Center for Endometriosis, Boston Children’s Hospital and Brigham and Women’s Hospital, Boston, Massachusetts, United States of America; 2 Department of Obstetrics, Gynecology, and Reproductive Biology, Brigham and Women’s Hospital and Harvard Medical School, Boston, Massachusetts, United States of America; 3 Division of Gynecology, Department of Surgery, Boston Children's Hospital and Harvard Medical School, Boston, Massachusetts, United States of America; 4 Department of Epidemiology, Harvard T.H. Chan School of Public Health, Boston, Massachusetts, United States of America; 5 Department of Obstetrics, Gynecology, and Reproductive Biology, College of Human Medicine, Michigan State University, Grand Rapids, Michigan, United States of America; 6 Division of Adolescent and Young Adult Medicine, Department of Medicine, Boston Children's Hospital and Harvard Medical School, Boston, Massachusetts, United States of America; University of Insubria, ITALY

## Abstract

Endometriosis is a painful gynecologic disease affecting one in ten reproductive aged women worldwide. Few studies have correlated this symptomatology with biomarker levels among women with and without endometriosis, and no studies correlating pain with biomarker levels have been performed in young patient populations. The purpose of this study was to examine whether CA125 correlates with different types and severity of pain among adolescents and young women with and without endometriosis and assess its performance as an endometriosis biomarker among those presenting with dysmenorrhea in this young population. Reproductive-aged women with laparoscopically-confirmed endometriosis (n = 282) and controls (n = 293) who participated in The Women’s Health Study: From Adolescence to Adulthood (A2A), a cohort of adolescents and young women enrolled from 2012–2018, were included in this cross-sectional analysis. Plasma CA125 values were measured using WERF EPHect compliant blood samples collected at enrollment. Average CA125 were calculated by self-reported pain type (i.e. dysmenorrhea, non-cyclic/general pelvic pain, dyspareunia), severity, and frequency in endometriosis cases and controls. Median age at blood draw was 24 years in controls and 17 years in cases, with 68% and 89% non-Hispanic white, respectively. Most endometriosis cases (95%) were rASRM stage I/II. Average CA125 values were 12.5 U/mL in controls and 12.1 U/mL in cases adjusted for age. CA125 did not differ by pain type, its severity, or frequency in endometriosis cases or controls. Among participants who reported dysmenorrhea, CA125 did not discriminate endometriosis cases from controls using cutoff of 35 U/mL (AUC = 0.51, 95%CI = 0.50–0.53). Among adolescents and young adult women, CA125 did not correlate with pain type. CA125 did not efficiently discriminate endometriosis cases from controls even when accounting for pain symptomatology. Average CA125 values were low in adolescents and young women in both endometriosis cases and controls, suggesting cautious interpretation may be needed when measuring CA125 in this population.

## Introduction

Endometriosis is a painful gynecologic disease affecting one in ten reproductive aged women worldwide [1]. Although over 50% of adults with endometriosis report onset of severe pelvic pain during adolescence, there is an average seven-year delay in diagnosis due to the current gold standard of surgical visualization for definitive diagnosis [2–4]. Presently, no reliable biomarkers exist for non-invasive diagnosis of endometriosis [5, 6]. Though many attempts have been made to identify and validate specific biomarkers, studies have been conducted mostly in adult populations limited by poor methodological quality including small sample sizes, lack of consideration of disease heterogeneity, and inappropriate control groups [6, 7].

Cancer Antigen125 (CA125) is a high molecular-weight glycoprotein normally expressed on tissues derived from the coelomic and mullerian epithelia including the uterus endometrium [8]. CA125 has been reported to be elevated in endometriosis patients and is the most commonly described, extensively studied endometriosis biomarker to date [6, 9–13]. A recent report suggested that CA125 may be predictive of endometriosis in symptomatic women with gynecological pain and/or subfertility [10]. However, this study was based on only 30 endometriosis cases and 28 surgical controls with heterogeneous endometriosis and control subtypes.

Patients with endometriosis have been shown to experience different levels of dysmenorrhea, non-cyclic/general pelvic pain, and dyspareunia, which are the most frequently reported pain outcomes among these women [9]. However, few studies have correlated this symptomatology with biomarker levels, and no studies correlating pain with biomarker levels have been performed in young patient populations. Endometriosis diagnosed in adolescence often presents with pain symptoms and superficial peritoneal lesions, which is often different from endometriosis diagnosed in adults [14]. Thus, it is important to understand biomarker performance in this population when aiming for earlier diagnosis of endometriosis.

The objective of this study was to examine whether CA125 correlates with different types and severity of pain among adolescents and young women with and without endometriosis using data collected at enrollment from the longitudinal cohort of the Women’s Health Study: From Adolescence to Adulthood (A2A).

## Material and methods

### Study population

The Women’s Health Study: From Adolescence to Adulthood (A2A) is a study of adolescents and women oversampled for those surgically diagnosed with endometriosis, enrolling participants from 2012 to 2018 [14]. Briefly, endometriosis cases were enrolled from Boston Children’s Hospital (BCH) and Brigham and Women’s Hospital (BWH) and were eligible if they were 1) female; 2) aged 7–55 years; and 3) had a surgical diagnosis of endometriosis. Controls at enrollment were selected through a combination of approaches that sampled the underlying population that gave rise to the cases [15]. Specifically, controls were women without a surgical diagnosis of endometriosis and were recruited from clinics at BCH and BWH and from the local Boston community through local advertisement, online postings, and/or word of mouth. At the time of enrollment, participants were emailed a link to a REDCap survey containing extensive questions assessing lifestyle and reproductive factors as well as level of pain, treatment regimen, and medication use. Participants who did not respond to the email link after three follow-up attempts were mailed a paper copy of the questionnaire. Annual questionnaires were collected via the same methods. All questionnaires collected after January 2014 were World Endometriosis Research Foundation Endometriosis Phenome and Biobanking Harmonization Project (WERF EPHect) compliant [16], which are available online (http://endometriosisfoundation.org/ephect). Clinical information for A2A participants undergoing surgery at BCH or BWH were collected using the WERF EPHect surgical form, including surgically visualized appearance of peritoneal lesions, endometrioma, and deep infiltrating disease and endometriosis revised American Society for Reproductive Medicine (rASRM) stage [17]. This study was approved by the Institutional Review Board of the BWH (Partners Human Research Committee; 2015P001101). All participants provided written consent for study participation, with parental consent plus participant assent for girls < 18 years.

### Pain assessment

Detailed information on symptoms of pain were obtained from the questionnaire completed by all A2A participants at enrollment of the study including whether the participant ever experienced pain with periods (dysmenorrhea), non-cyclic/general pelvic pain (pain not associated with menses), and dyspareunia in their lifetime [16]. Severity of dysmenorrhea, general pelvic pain, and dyspareunia was assessed using the visual analogue scale which is an 11-point numeric pain rating scale with 0 = no pain and 10 = worst imaginable pain [18]. Additionally, information on frequency of dysmenorrhea, general pelvic pain, and dyspareunia was collected.

### Blood collection

Blood samples were collected at enrollment in compliance with the standardized tools of WERF EPHect [16, 17, 19, 20]. At the time of sample collection, participants completed a biospecimen questionnaire on which they reported date of last menstrual period, timing of last foods/beverages consumed, and recent medication and hormone use. Blood samples were collected, processed into plasma, serum and buffy coats, and stored at ≤-80°C per WERF EPHect fluids standard operating protocols [20].

### CA125 measurement

CA125 was measured in 100μL of plasma using a Food and Drug Administration (FDA)-approved clinical chemiluminescent immunoassay (CA125 II) at the Clinical and Epidemiologic Laboratory at Boston Children’s Hospital. The assay reproducibility for CA125 on the E170 automated instrument was high with coefficient of variation in blinded duplicate samples of 1.15% [21].

### Covariates

Information on age, race, body mass index (BMI; kg/m^2^), smoking status, age at menarche, menstrual cycle phase at time of blood draw, hormonal medication and analgesic use within 30 days from blood draw were abstracted from the questionnaire assessed at enrollment and/or biospecimen questionnaire data. For women aged ≥20 years, BMI was categorized according to the World Health Organization Criteria: underweight (BMI < 18.5 kg/m^2^), normal weight (BMI 18.5–24.9 kg/m^2^), overweight (BMI 25–29.9 kg/m^2^), or obese (BMI ≥ 30 kg/m^2^). For those <20 years, the age- and gender-specific BMI Z-score was calculated, and participants were categorized as underweight (Z-score ≤ −2), normal weight (Z-score >−2 to <1), overweight (Z-score 1–2), or obese (Z-score > 2). For endometriosis cases, information on rASRM stage and endometriosis subtype at surgery closest to blood draw was abstracted from the WERF-EPHect surgical form and self-reported age at first endometriosis symptoms was abstracted from the questionnaire obtained at enrollment of the study.

### Statistical analysis

Among the 656 participants (347 cases and 306 controls) who had a CA125 measurement for their blood samples collected at enrollment, we excluded participants who did not complete the questionnaire at enrollment (n = 13), completed the questionnaire at enrollment more than 60 days before/after their blood draw at enrollment (n = 59), were enrolled as a control at enrollment, but were diagnosed with endometriosis within 2 years after enrollment (n = 1), and were premenarchal at enrollment (n = 5) for a final analytic sample size of 282 surgically confirmed endometriosis cases and 293 controls.

CA125 values were log-transformed to improve normality. We calculated the age-adjusted geometric means and 95% confidence intervals (CIs) of CA125 for all covariates and pain variables described above. Participants were excluded from analyses where they were missing the main exposure. Trend tests were calculated by modeling the exposure variable categories as ordinal adjusted for continuous age. Logistic regression analysis was used to calculate the Area Under the Curve (AUC) and 95% CIs in discriminating endometriosis cases from controls among participants who reported dysmenorrhea by pain levels using thresholds of 30 U/mL and 35 U/mL. All statistical analyses were performed using SAS version 9.4 (SAS Institute Inc., Cary, NC).

## Results

In total, 575 participants, including 282 laparoscopically-confirmed endometriosis cases and 293 controls, were included in the analyses (Table 1). Median age at blood draw was 24 years in controls and 17 years in cases, with 68% and 89% non-Hispanic white, respectively. Most (81%) of the cases were using hormonal medication at blood draw compared to only 46% of the controls. The majority of cases (95%) were rASRM stage I or II. Overall, the average CA125 was 14.4 U/mL ranging from 3.6 to 117.8 U/mL.

**Table 1:** Association between demographic/reproductive factors and circulating CA125 among surgically confirmed endometriosis cases and controls^a^.

	Endometriosis (n = 282)		No Endometriosis (n = 293)	
Characteristics	N (%)	CA125 (U/mL)^b^	N (%)	CA125 (U/mL)^b^
Overall	282	12.1 (11.4, 12.8)	293	12.5 (11.8, 13.3)
Age at blood draw				
<18 years old	148 (52.5)	11.9 (11.1, 12.8)	10 (3.4)	12.5 (9.1, 17.4)
18 to < 22 years old	65 (23.1)	12.7 (11.3, 14.2)	58 (19.8)	11.9 (10.4, 13.6)
22 to < 26 years old	25 (8.9)	9.8 (8.2, 11.8)	122 (41.6)	12.0 (10.9, 13.1)
26 to< 30 years old	19 (6.7)	12.7 (10.3, 15.6)	51 (17.4)	13.7 (11.8, 15.8)
30+ years old	25 (8.9)	13.6 (11.4, 16.3)	52 (17.8)	13.5 (11.7, 15.6)
Race (RACE2)				
White	251 (89.0)	12.4 (11.7, 13.1)	198 (68.3)	12.4 (11.5, 13.3)
Non-white	31 (11.0)	10.0 (8.5, 11.7)	92 (31.7)	12.9 (11.5, 14.3)
Body Mass Index (kg/m^2^)^c^				
Underweight	4 (1.4)	11.6 (7.4, 18.3)	10 (3.4)	9.4 (6.8, 13.1)
Normal weight	167 (59.6)	12.5 (11.7, 13.5)	197 (67.5)	12.5 (11.6, 13.5)
Overweight	60 (21.4)	11.6 (10.3, 13.1)	52 (17.8)	12.9 (11.2, 14.9)
Obese	49 (17.5)	11.5 (10.1, 13.0)	33 (11.3)	12.8 (10.7, 15.3)
Smoking status				
Never	254 (93.7)	12.1 (11.4, 12.8)	268 (91.8)	12.6 (11.9, 13.4)
Former	14 (5.2)	12.0 (9.3, 15.5)	18 (6.2)	11.3 (8.7, 14.5)
Current	3 (1.1)	9.5 (5.6, 16.2)	6 (2.1)	9.5 (6.2, 14.4)
Age at menarche				
≤ 10 years	50 (17.8)	11.1 (9.8, 12.7)	34 (11.6)	11.8 (9.9, 14.1)
11–12 years	147 (52.3)	12.5 (11.6, 13.5)	135 (46.2)	12.7 (11.6, 13.9)
13–14 years	78 (27.8)	11.7 (10.5, 13.0)	111 (38.0)	12.3 (11.1, 13.5)
>14 years	6 (2.1)	15.8 (10.9, 22.9)	12 (4.1)	13.9 (10.3, 18.7)
Menstrual cycle phase at blood draw^d^				
Early follicular	6 (17.7)	12.1 (7.3, 19.9)	24 (15.8)	17.3 (14.0, 21.3)
Late follicular	5 (14.7)	17.5 (10.2, 30.0)	24 (15.8)	13.3 (10.8, 16.4)
Peri-ovulation	2 (5.9)	9.0 (3.8, 21.3)	10 (6.6)	12.3 (8.9, 17.0)
Luteal	8 (23.5)	14.5 (9.5, 22.2)	41 (27.0)	13.9 (11.9, 16.4)
Long cycles	9 (26.5)	15.3 (10.1, 23.1)	37 (24.3)	15.1 (12.8, 17.9)
Irregular cycles	4 (11.8)	12.8 (7.0, 23.3)	16 (10.5)	11.9 (9.2, 15.4)
Hormonal medication use within past 30 days at blood draw				
Not using hormones	39 (19.1)	13.7 (11.8, 15.9)	155 (53.6)	14.1 (13.0, 15.3)
Using hormones	165 (80.9)	12.0 (11.1, 12.8)	134 (46.4)	10.9 (10.0, 11.9)
Analgesic use within past 30 days at blood draw				
Did not use any pain medication	37 (19.8)	11.4 (9.8, 13.3)	155 (60.1)	12.0 (11.1, 13.0)
Used any pain medication	150 (80.2)	12.1 (11.2, 13.0)	103 (39.9)	12.7 (11.5, 14.0)
**Endometriosis-specific**	** **	** **	** **	** **
ASRM Stage				
Stage I/II	246 (95.4)	11.6 (11.0, 12.3)	N/A	N/A
Stage III/IV	12 (4.7)	21.2 (16.4, 27.5)	N/A	N/A
Endometriosis subtype				
Superficial peritoneal lesion only	256 (95.9)	11.7 (11.1, 12.3)	N/A	N/A
Endometrioma	5 (1.9)	51.7 (35.7, 74.9)	N/A	N/A
Deep infiltrating	5 (1.9)	12.2 (8.5, 17.5)	N/A	N/A
Endometrioma and Deep infiltrating	1 (0.4)	37.1 (16.3, 84.7)	N/A	N/A
Age at first endometriosis symptoms				
≤ 12 years	87 (34.4)	12.7 (11.4, 14.0)	N/A	N/A
13 years	51 (20.2)	11.1 (9.7, 12.6)	N/A	N/A
14–15 years	74 (29.3)	12.1 (10.9, 13.5)	N/A	N/A
≥ 16 years	41 (16.2)	12.3 (10.6, 14.3)	N/A	N/A

^a^Number of missings for characteristic variables: BMI (n = 3), smoking status (n = 12), race (n = 3), age at menarche (n = 2), menstrual cycle phase at blood draw (n = 90), hormonal medication use at time of blood draw (n = 82), Analgesic use at time of blood draw (n = 130), ASRM stage (n = 24), age at first endometriosis symptoms (n = 29).

^b^Geometric mean (95%CI) adjusted for age (continuous).

^c^For women aged ≥20 years: underweight (BMI < 18.5 kg/m^2^), normal weight (BMI 18.5–24.9 kg/m^2^), overweight (BMI 25–29.9 kg/m^2^), or obese (BMI ≥ 30 kg/m^2^) according to World Health Organization criteria; For those <20 years, the age- and gender-specific BMI Z-score was calculated, and participants were categorized as underweight (Z-score ≤ −2), normal weight (Z-score >−2 to <1), overweight (Z-score 1–2), or obese (Z-score > 2).

^d^Among participants not on hormones at the time of blood draw.

The average CA125 values were 12.5 (95%CI = 11.8–13.3) U/mL in controls and 12.1 (95%CI = 11.4–12.8) U/mL in cases (p = 0.96), and the association between demographic characteristics and CA125 values were similar in general regardless of disease status (Table 1). Compared to those over age 30, younger age at blood draw was suggestively associated with lower CA125 values. Current smokers had lower CA125 values compared to former or never smokers, although there were only nine current smokers. Participants whose age at menarche was greater than 14 had higher CA125 values on average compared to those whose menarche started at a younger age. CA125 values were higher when blood was drawn during the follicular phase compared to other menstrual cycle phases. Regardless of whether the participants were diagnosed of endometriosis, those who were on hormonal medication at blood draw had lower CA125 values compared to those who were not. Analgesic use at time of blood draw was not associated with CA125 values. Among endometriosis cases, those diagnosed with advanced stage had a higher CA125 compared to those diagnosed with early stage disease. When we examined levels by endometriosis subtypes, those with endometrioma had the highest average CA125 values (51.7, 95%CI = 35.7–74.9 U/mL), although the sample size was limited to five cases. Endometriosis cases with superficial peritoneal lesions only (n = 256) and those with deep infiltrating endometriosis lesions only (n = 5) had lower CA125 values (11.7, 95%CI = 11.1–12.3 U/mL; 12.2, 95%CI = 8.5–17.5 U/mL respectively). Age at first endometriosis symptom was not associated with CA125.

We examined the association between symptoms of pain type, severity, duration and CA125 values in endometriosis cases and controls (Table 2). Overall, average CA125 values were similar across participants’ self-reported symptoms of ever experiencing dysmenorrhea, general pelvic pain, or dyspareunia. We did not observe a correlation between severity and frequency of these pain types and CA125 values among endometriosis cases or controls (p-trend >0.05). Since CA125 is strongly influenced by hormonal medication, we examined the association between pain symptoms and CA125 values among controls who were not on hormonal medication at time of blood draw (S1 Table). Controls reporting severe general pelvic pain was associated with higher CA125 (16.3, 95%CI = 11.3–23.6 U/mL) compared to those with mild general pelvic pain (13.4, 95%CI = 9.0–19.9 U/mL) although the trend was not statistically significant (p-value = 0.40). Interestingly, controls who reported never experiencing dyspareunia had higher CA125 (15.3, 95%CI = 13.5–17.4 U/mL) compared to those who ever experienced dyspareunia (13.6, 95%CI = 11.4–16.3 U/mL), with a significant trend by severity within 24 hours after vaginal intercourse (p-trend = 0.04). However, since there were only five control participants who reported severe dyspareunia, this observation may be due to chance.

**Table 2:** Association between pain symptoms and circulating CA125 among surgically confirmed endometriosis cases and controls^a^.

	Endometriosis (n = 282)		No Endometriosis (n = 293)	
	N (%)	CA125 (U/mL)^b^	N (%)	CA125 (U/mL)^b^
**Dysmenorrhea**	** **	** **	** **	** **
Ever experienced period pain	** **	** **	** **	** **
Never	6 (2.1)	12.4 (8.5, 18.0)	52 (17.8)	12.3 (10.6, 14.1)
Ever	276 (97.9)	12.1 (11.4, 12.8)	241 (82.3)	12.6 (11.8, 13.4)
p-value		0.89		0.76
Severity of period pain^c^^,^^d^				
Mild	10 (4.0)	11.9 (8.9, 15.9)	62 (28.3)	12.3 (10.7, 14.0)
Moderate	25 (10.1)	12.1 (10.1, 14.6)	97 (44.3)	11.9 (10.7, 13.3)
Severe	213 (85.9)	12.2 (11.4, 13.0)	60 (27.4)	13.3 (11.6, 15.2)
p-trend		0.90		0.23
Frequency of period pain^c^^,^^e^				
Never/rarely	7 (4.4)	12.3 (8.8, 17.2)	14 (6.8)	13.6 (10.4, 17.7)
Occasionally	6 (3.8)	18.4 (12.8, 26.5)	72 (35.1)	10.7 (9.5, 12.0)
Often	6 (3.8)	15.2 (10.6, 21.7)	39 (19.0)	13.7 (11.7, 16.0)
Usually	14 (8.8)	12.0 (9.5, 15.2)	34 (16.6)	11.5 (9.7, 13.6)
Always	127 (79.4)	11.9 (11.0, 12.8)	46 (22.4)	14.0 (12.1, 16.2)
p-trend		0.12		0.04
**General Pelvic Pain**	** **	** **	** **	** **
Ever experienced general pelvic pain				
Never	91 (32.9)	12.0 (10.9, 13.3)	233 (79.8)	12.4 (11.6, 13.3)
Ever	186 (67.2)	12.1 (11.3, 12.9)	59 (20.2)	12.9 (11.3, 14.8)
p-value		0.94		0.58
Severity of general pelvic pain ^d^^,^^f^				
Mild	12 (7.6)	14.5 (11.2, 18.8)	10 (25.6)	12.2 (8.8, 17.0)
Moderate	29 (18.4)	11.0 (9.3, 13.0)	13 (33.3)	11.6 (8.7, 15.5)
Severe	117 (74.1)	12.3 (11.3, 13.3)	16 (41.0)	13.3 (10.2, 17.2)
p-trend		0.77		0.51
Frequency of general pelvic pain ^e^^,^^f^				
2–3 days/month or fewer	27 (28.4)	12.3 (10.5, 14.5)	25 (73.5)	11.7 (9.5, 14.6)
1–6 days per week	39 (41.1)	12.1 (10.6, 13.9)	9 (26.5)	14.3 (10.0, 20.5)
Every day	29 (30.5)	11.9 (10.2, 14.0)	0 (0.0)	N/A
p-trend		0.81		N/A
**Dyspareunia**	** **	** **	** **	** **
Ever experienced dyspareunia^g^				
Never	22 (20.6)	12.7 (10.1, 15.9)	132 (61.1)	13.0 (11.9, 14.2)
Ever	85 (79.4)	12.0 (10.6, 13.4)	84 (38.9)	12.2 (10.9, 13.7)
p-value		0.67		0.39
Severity of dyspareunia within 24 hours after vaginal intercourse/penetration^d^^,^^e^^,^^g^^,^^h^				
Mild	26 (44.8)	13.8 (11.2, 16.9)	149 (85.6)	12.5 (11.5, 13.6)
Moderate	15 (25.9)	12.8 (9.7, 16.8)	17 (9.8)	15.1 (11.9, 19.2)
Severe	17 (29.3)	10.7 (8.4, 13.8)	8 (4.6)	9.5 (6.7, 13.5)
p-trend		0.15		0.12
Frequency of dyspareunia^g^^,^^i^				
Occasionally	21 (25.0)	11.8 (9.2, 15.1)	53 (69.7)	11.9 (10.4, 13.7)
Often	17 (20.2)	11.4 (8.7, 14.9)	10 (13.2)	11.3 (8.2, 15.6)
Usually	21 (25.0)	12.9 (10.1, 16.5)	11 (14.5)	13.9 (10.1, 19.0)
Always	25 (29.8)	11.6 (9.3, 14.5)	2 (2.6)	6.2 (3.0, 12.8)
p-trend		0.79		0.08

^a^Number of missings for pain variables: severity of period pain (n = 12), frequency of period pain (n = 14), ever experienced general pelvic pain (n = 6), severity of general pelvic pain (n = 11), ever dyspareunia (n = 1), severity of dyspareunia (n = 7), frequency of dyspareunia (n = 9).

^b^Geometric mean (95%CI) adjusted for age (continuous).

^c^Among participants who experienced period pain within the last 12 months.

^d^Severity of pain was categorized based on the VAS scale: Mild (1–3), Moderate (4–6), Severe (7–10).

^e^Among participants who answered the WERF EPHect version of the questionnaire.

^f^Among participants who experienced general pelvic pain within the last 3 months.

^g^Among participants aged 18 or older and reported ever having vaginal intercourse/penetration.

^h^Among participants who reporting having dyspareunia at their current age range (16–20 years, 21–30 years, 31–40 years, 41+ years).

^i^Among participants who reported having dyspareunia in the last 12 months.

**Fig 1:**
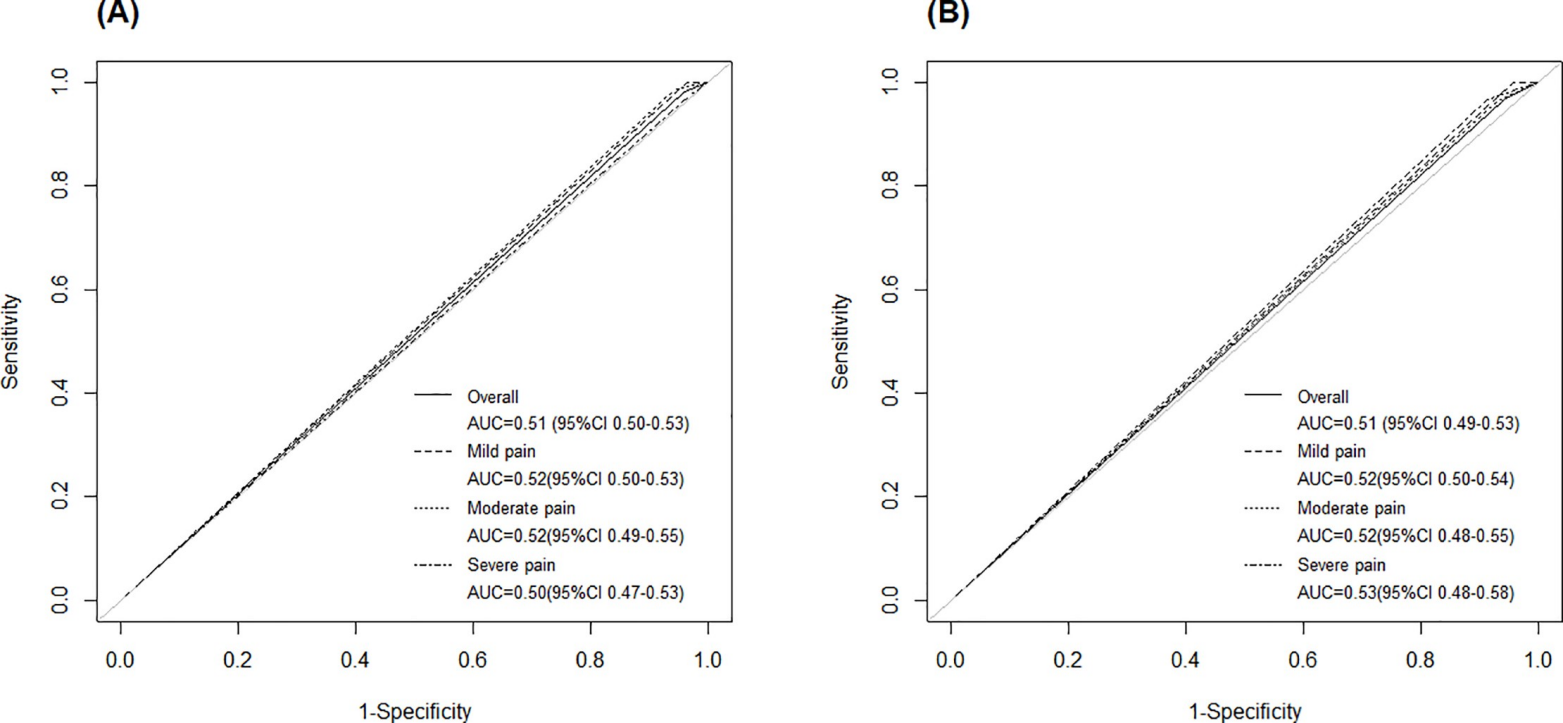
Performance of CA125 in discriminating endometriosis cases from controls among participants presenting with dysmenorrhea in the adolescence to adulthood study. **(A)** Receiver Operating Curves (ROC) using CA125 cutoff of 35 U/mL and **(B)** using CA125 cutoff of 30 U/mL among participants presenting with dysmenorrhea (overall = solid line; mild pain = dashed line; moderate pain = dotted line; severe pain = dash-dotted line).

Among participants with self-reported dysmenorrhea at baseline, we evaluated the performance of CA125 in discriminating endometriosis cases from controls overall and by level of pain severity (Fig 1). Overall, CA125 did not perform well using the clinical cutpoint of 35 U/mL (AUC = 0.51, 95%CI: 0.50–0.53) or 30 U/mL (AUC = 0.51, 95%CI: 0.49–0.53). When examining the performance of CA125 by the level of pain severity (mild, moderate, severe), the performance did not differ in discriminating endometriosis cases from controls using both cutpoints of 35 U/mL and 30 U/mL with AUCs ranging from 0.50 to 0.53.

## Discussion

In this young population where more than 80% of the participants are age <30, we observed that circulating CA125 levels were not correlated with different pain types and severity in both laparoscopically-confirmed endometriosis cases and controls. We also showed that CA125 did not perform well discriminating endometriosis cases from controls in young women presenting with dysmenorrhea.

To our knowledge, this is the first study reporting the correlation between different types and severity of pain and CA125 levels in young women with and without endometriosis. Emerging evidence suggests pain is a heterogeneous, multifaceted condition [22], and therefore it is plausible that molecular features may differ by different pain presentations. While we did not observe significant association between pain and CA125, one small study (n = 69) observed higher CA125 in endometriosis patients with severe dysmenorrhea and dyspareunia compared to patients with no pain, although the association was not statistically significant [23]. This inconsistent observation may be in part due to more than 80% of our endometriosis cases using hormonal medication at blood draw, resulting in lower CA125 and attenuating the CA125 differences by pain types and severity. In addition, this inconsistency may also be due to differences in age groups, clinical presentation, and differences in the definition of “pain severity”, since pain severity could be defined in multiple ways (e.g. severity on average over the life course, severity in the past 12 months) between our study and the prior publication. Furthermore, CA125 values could have been influenced by different therapeutic strategies which are available to treat endometriosis other than hormone therapy, such as differences in surgical procedures, phytotherapy with the use of medicinal plants, and supplementation [24–26]. We did not observe a significant association between recent analgesic use and CA125, however, the associations between common medication use and CA125 is still limited.

In our study, CA125 did not perform well in discriminating endometriosis cases from controls in young women. Although CA125 has consistently been reported to be associated with endometriosis, many prior studies combined patients with diverse clinical phenotypes and categorized endometriosis as one disease [5, 6, 9]. In fact, recent studies support that multiple molecular mechanisms are involved in endometriosis pathogenesis, including the complex genetic nature and epigenetic mechanisms influencing hormonal, immunologic, and inflammatory aberrations in endometriosis patients [27, 28]. The heterogeneous presentation and molecular mechanisms underlying endometriosis suggest different etiological pathways by subtypes and combining these subtypes into a single endometriosis definition in prior studies may have thwarted progress in finding a reproducible endometriosis biomarker, as a clear signal with any given subtype being overwhelmed by the differences between subtypes [17].

While we did not observe improved discriminatory performance in CA125 after accounting for severity of dysmenorrhea, there have been few studies that reported CA125 performed well in discriminating endometriosis cases from those without among women with pain symptoms [10, 23, 29]. This inconsistent observation could be due in part to the differences in endometriosis phenotype and age range of the study participants. Prior studies reported CA125 does not perform as well in detecting minimal to mild endometriosis (rASRM stage I/II) [30–32], which is known to be the predominant clinical phenotype of adolescent endometriosis [14, 33, 34]. Furthermore, the average age of endometriosis cases in one prior study was 34 while the average age in our endometriosis cases was 19.6. Therefore, CA125 values measured in adolescents and young women may need to be interpreted differently with more caution.

To our knowledge, this is the first study that reported demographic characteristics in relation to CA125 values in healthy adolescents and young women. CA125 is known to be influenced by personal characteristics in healthy individuals [12, 35–38], and only two studies have examined correlates of CA125 in premenopausal women, with the youngest age category being women age <30 years [12, 35]. Consistent with prior studies, we observed that current smoking and hormone use at blood draw was associated with lower CA125, and blood draw at follicular phase was associated with higher CA125 in controls. Interestingly, young women in their late teens and early 20s had lower CA125 compared to those age 30 or older, suggesting we may need to consider lower thresholds when interpreting CA125 values among these healthy young women.

The major strength of our study is the large sample of adolescents and young women with laparoscopically-diagnosed endometriosis and use of controls sampled from the underlying population that gave rise to the cases. Our study participants were also well annotated for their pain symptoms and had valid biomarker measurements using the WERF EPHect compliant questionnaires and biospecimens. However, due to the case-control design of the study, symptoms of pain may have been recalled differently in cases compared to controls, which may have led to over or underestimation of the association. Since most of the participants were on hormonal medication at time of blood draw, we were not able to stratify all our analyses by use of hormonal medication, which is known to influence CA125 values. This may have limited the ability to detect the association between different symptoms of pain and CA125 values. However, given that many young women currently use hormonal medication, we believe our current results are generalizable. Moreover, the clinical utility of CA125 in tailoring treatment in women with pelvic pain is still unclear. Our study consisted primarily of a white population and was based in a specific region of the United States, which may limit the generalizability of our study results.

## Conclusion

Our analyses suggest that CA125 values do not correlate with types of pain (i.e. dysmenorrhea, acyclic pain, and dyspareunia), or its severity or frequency among adolescents and young adult women. CA125 did not efficiently discriminate endometriosis cases from controls using the clinical cutpoints of 35 U/mL or 30 U/mL even when accounting for pain symptomatology in this young population. Average blood CA125 values were low in adolescents and young women in both endometriosis cases and controls, suggesting that cautious interpretation may be needed when measuring CA125 in adolescents and young women.

## Supporting information

S1 Table Association between pain symptoms and circulating CA125 among controls not on hormones at time of blood draw. (DOCX)
