# Supplementary material for: Evaluation of CA125 in relation to pain symptoms among adolescents and young adult women with and without surgically-confirmed endometriosis
Source: PLoS One. 2020 Aug 24;15(8):e0238043. doi: 10.1371/journal.pone.0238043 (PMC7444809; doi:10.1371/journal.pone.0238043)
Supplement: S1 Table — (DOCX) [file pone.0238043.s001.docx]

| **Supplemental Table 1. Association between pain symptoms and circulating CA125 among controls not on hormones at time of blood draw^a^** | | |
| --- | --- | --- |
|  |  |  |
|  | **Controls not on hormones (n=155)** | |
|  | **N (%)** | **CA125 (U/mL)^b^** |
| **Dysmenorrhea** |  |  |
| Ever experienced period pain |  |  |
| Never | 27 (17.4) | 13.2 (10.8, 16.1) |
| Ever | 128 (82.6) | 14.3 (13.1, 15.7) |
| p-value | 0.47 |  |
| Severity of period pain ^c,d^ |  |  |
| Mild | 25 (20.7) | 15.7 (12.7, 19.5) |
| Moderate | 54 (44.6) | 13.0 (11.3, 15.0) |
| Severe | 42 (34.7) | 14.8 (12.5, 17.4) |
| p-trend | 0.50 |  |
| Frequency of period pain ^c,e^ |  |  |
| Never/rarely | 3 (2.9) | 16.1 (8.9, 29.1) |
| Occasionally | 28 (27.2) | 12.0 (9.9, 14.6) |
| Often | 21 (20.4) | 15.1 (12.1, 18.9) |
| Usually | 19 (18.5) | 13.0 (10.3, 16.5) |
| Always | 32 (31.1) | 16.0 (13.4, 19.2) |
| p-trend | 0.10 |  |
| **General Pelvic Pain** |  |  |
| Ever experienced general pelvic pain |  |  |
| Never | 120 (77.9) | 13.9 (12.6, 15.3) |
| Ever | 34 (22.1) | 14.8 (12.4, 17.7) |
| p-value | 0.54 |  |
| Severity of general pelvic pain ^d,f^ |  |  |
| Mild | 7 (30.4) | 13.4 (9.0, 19.9) |
| Moderate | 8 (34.8) | 13.4 (9.2, 19.4) |
| Severe | 8 (34.8) | 16.3 (11.3, 23.6) |
| p-trend | 0.40 |  |
| Frequency of general pelvic pain^e,f^ |  |  |
| 2-3 days/month or fewer | 15 (75) | 12.5 (9.6, 16.4) |
| 1-6 days per week | 5 (25) | 22.3 (13.9, 35.6) |
| Every day | N/A | N/A |
| p-trend | N/A | N/A |
| **Dyspareunia** |  |  |
| Ever dyspareunia^g^ |  |  |
| Never | 69 (66.4) | 15.3 (13.5, 17.4) |
| Ever | 35 (33.7) | 13.6 (11.4, 16.3) |
| p-value | 0.29 |  |
| Severity of dyspareunia within 24 hours after vaginal intercourse/penetration^d,e,g,h^ |  |  |
| Mild | 66 (83.5) | 15.2 (13.4, 17.2) |
| Moderate | 8 (10.1) | 14.4 (9.9, 20.7) |
| Severe | 5 (6.3) | 9.0 (5.6, 14.3) |
| p-trend | 0.04 |  |
| Frequency of dyspareunia^g,i^ |  |  |
| Occasionally | 22 (75.9) | 12.5 (10.1, 15.5) |
| Often | 2 (6.9) | 23.4 (11.5, 47.6) |
| Usually | 3 (10.3) | 15.0 (8.1, 27.9) |
| Always | 2 (6.9) | 6.7 (3.3, 13.8) |
| p-trend | 0.09 |  |
| ^a^Number of missings for pain variables: severity of period pain (n=3), frequency of period pain (n=1), ever experienced general pelvic pain (n=1), severity of general pelvic pain (n=1), ever dyspareunia (n=1), severity of dyspareunia (n=0), frequency of dyspareunia (n=6) | | |
| ^b^Geometric mean (95%CI) adjusted for age (continuous) | | |
| ^c^Among participants who experienced period pain within the last 12 months | | |
| ^d^Severity of pain was categorized based on the VAS scale: Mild (1-3), Moderate (4-6), Severe (7-10) | | |
| ^e^Among participants who answered the WERF EPHect version of the questionnaire | | |
| ^f^Among participants who experienced general pelvic pain within the last 3 months | | |
| ^g^Among participants aged 18 or older and reported ever having vaginal intercourse/penetration | | |
| ^h^Among participants who reporting having dyspareunia at their current age range (16-20 yrs, 21-30 yrs,31-40 yrs, 41+ yrs) | | |
| ^i^Among participants who reported having dyspareunia in the last 12 months | | |
